# Characteristics and correlations of leaf stomata in different *Aleurites montana* provenances

**DOI:** 10.1371/journal.pone.0208899

**Published:** 2018-12-18

**Authors:** Tao Hong, Han Lin, Dongjin He

**Affiliations:** 1 Forestry College, Fujian Agriculture and Forestry University, Fuzhou, Fujian, the People's Republic of China; 2 Fujian Yongan Forestry(Group)Joint-Stock CO., LTD., Yongan, Fujian, the People's Republic of China; Fred Hutchinson Cancer Research Center, UNITED STATES

## Abstract

Stomata are important indexes in studies of plant origin, evolution, and classification and are important organs in plant phylogenetic relationship studies. Nine *Aleurites montana* provenances were used in this study to examine stomatal density, stomatal area, stomatal perimeter, long axis length, and short axis length. The correlation and cluster analyses were conducted among the morphological parameters of the pores. The results showed that there were significant differences in different *A*. *Montana* provenances in terms of stomatal morphology parameters. The average stomatal density, stomatal area, stomatal perimeter, stomatal long axis length, and stomatal short axis length of each provenance was between 224.16–307.10 stomata/mm^2^, 80.42–99.76 μm, 36.39–41.32 μm, 15.89–18.44 μm, and 6.53–7.46 μm, respectively, and the coefficient of variation was between 9.18%–20.15%, 17.57%–33.77%, 9.31%–18.79%, 9.71%–18.48%, and 10.26%–21.57%, respectively. Correlation analysis shows that there was a significant negative correlation between stomatal density and stomatal shape parameters (stomatal area, stomatal perimeter, stomatal long axis, stomatal short axis) and there was a significant positive correlation between stomatal parameters. There was no significant correlation between geographical environment factors and stomatal characteristics. There is a close relationship between stomatal morphology and stomatal conductance whereby dense small stomata can quickly adapt to changes in the environment; considering the characteristics *A*. *montana* stomata in terms of stomatal stability and ability to adapt to the environment, the Jianyang, Zhenghe, Fuding, Shaxian provenances were identified as being more suitable for planting at different sites. This study provides a theoretical basis for the genetic improvement and breeding of high quality *A*. *montana* provenances.

## Introduction

Stomata are the main channels for plants to exchange water and gas with the environment within the main photosynthetic organs and are closely correlated with plant physiological activities such as photosynthesis, respiration, and transpiration [1,2]. Stomata are easily affected by external environmental conditions. The micromorphological characteristics of plant leaves have been commonly used for plant classification [3,4]. Leaf stomata in different plants possess significantly different characteristics such as density, size, and shape [5]; stomata in plants from the same genus or from different germplasm resources of the same species also differ [6,7]. Plant epidermis stomata characteristics represent an important area in plant germplasm resource diversity studies. Stomata are important organs in plant phylogenetic relationship studies and their characteristics are important indexes in studies of plant origin, evolution, and classification [8,9,10].

*Aleurites montana* is a genus of deciduous trees in the *Euphorbiaceae*. As a native tree species with high economic value, *A*. *montana* has been cultivated for more than 1000 years in southern parts of China, with cultivation mainly distributed in Guangdong, Guangxi, Fujian, Jiangxi, Zhejiang, Taiwan, Yunnan, and Guizhou provinces [11]. The straight trunk of *A*. *montana* makes it a satisfactory building and furniture production material. Furthermore, *A*. *montana* is also a good material for edible fungi cultivation. Dry seeds of *A*. *montana* contain 60–70% oil; this tung oil is a promising raw material for new environmental chemical products and could be used in areas such as agriculture, industry, fishery, medicine, and military, and as a promising biological substitute for diesel [12,13]. Thus, regional *A*. *montana* provenance tests have been conducted by our group since 2008 [14,15].

Studies on *A*. *montana* mainly focused on seedling growth, seedling physiological indexes, and exploration and utilization of biological resources. Studies comparing *A*. *montana* from different regions have previously focused on leaf characteristics and chlorophyll fluorescence [14,15]. Studies of stomatal characteristics in different provenances have not, however, previously been reported. In this study, the characteristics of foliar surfaces were examined in nine *A*. *montana* provenances from Fujian Province using a stereo microscope. Stomatal characteristic differences were analyzed and cluster analysis was conducted in the nine *A*. *montana* provenances to provide a basis for *A*. *montana* resource utilization.

## Materials and methods

### Test areas

Nine *A*. *montana* provenances were used with seeds selected from Fu’an, Fuding, Jiaocheng, Putian, Shaxian, Shunchang, Youxi, Jianyang, and Zhenghe in 2008 used to generate seedlings (Table 1). For provenance tests, one-year-old seedlings were planted in the Jiaoxi forest farm (Jianyang) that is located in the northwest of Fujian Province and south Wuyi Mount at the geographic coordinates 118°21´35″E and 27°06´20″N. Located in the subtropical monsoon climate zone, the annual temperature difference of this test field is small, with higher temperatures and more precipitation during summer and a generally warm and humid climate during autumn and winter. The test area is heavily forested with a variety of plants.

**Table 1:** Site information for *Aleurites montana* provenances.

Species	Longitude	Latitude	Rainfall/ml	Temperature/°C
FA	119.43	26.83	1646.3	19.3
FD	120.01	26.89	1661.6	18.5
JC	119.15	26.46	1949.1	19.8
PT	119.25	25.78	1523.2	20.2
SX	117.84	26.39	1657.7	19.1
SC	117.75	26.81	1691.4	18.5
YX	118.15	26.14	1603.6	18.9
JY	118.15	27.04	1696.5	18.1
ZH	118.94	27.36	1624.3	18.3

FA-Fuan, FD-Fuding, JC-Jiaocheng, PT-Putian, SX-Shaxian, SC-Shunchang, YX-Youxi, JY-Jianyang, ZH-Zhenghe.

### Material collection and analysis

#### Sampling plots and material collection

Provenance tests were conducted in random blocks; a total of 12 blocks were set with each block consisting of nine minor blocks of 100 m^2^ that contained 25 *A*. *montana* from each provenance, respectively, and were separated by isolation belts [16]. *A*. *montana* leaves from different provenances were collected in late September 2017. Three normally growing sample plants from each provenance were selected and three pieces of healthy mature leaves on the lower part of newly grown branches were collected from each plant. Five replicates were taken for each sample. In total, 45 leaves were collected for each provenance and the collected leaves were preserved immediately in formalin–acetic acid–alcohol (FFA) fixative solution.

#### Stomata slice making

For the same plants, leaf stomata characteristics such as density and size were determined. These factors are known to be affected by maturity of the leaf [17,18,19], leaf position in plant [20,21], and stoma position in leaf [22]. Therefore, leaves of similar size and maturity were used to avoid experimental errors. Leaves were removed from the FAA fixative solution and washed using distilled water. Small leaf segments were then taken from the middle part of these leaves near the vein (adaxial sides) and placed in 20% sodium hypochlorite solution for segregation. After turning translucent, leaf segments were removed from the segregation solution, washed on slides, and their epidermis was isolated. The isolated epidermis was stained by sarranine for 1 min, washed with distilled water, and then sealed using neutral balsam.

#### Stomata observation

**Fig 1:**
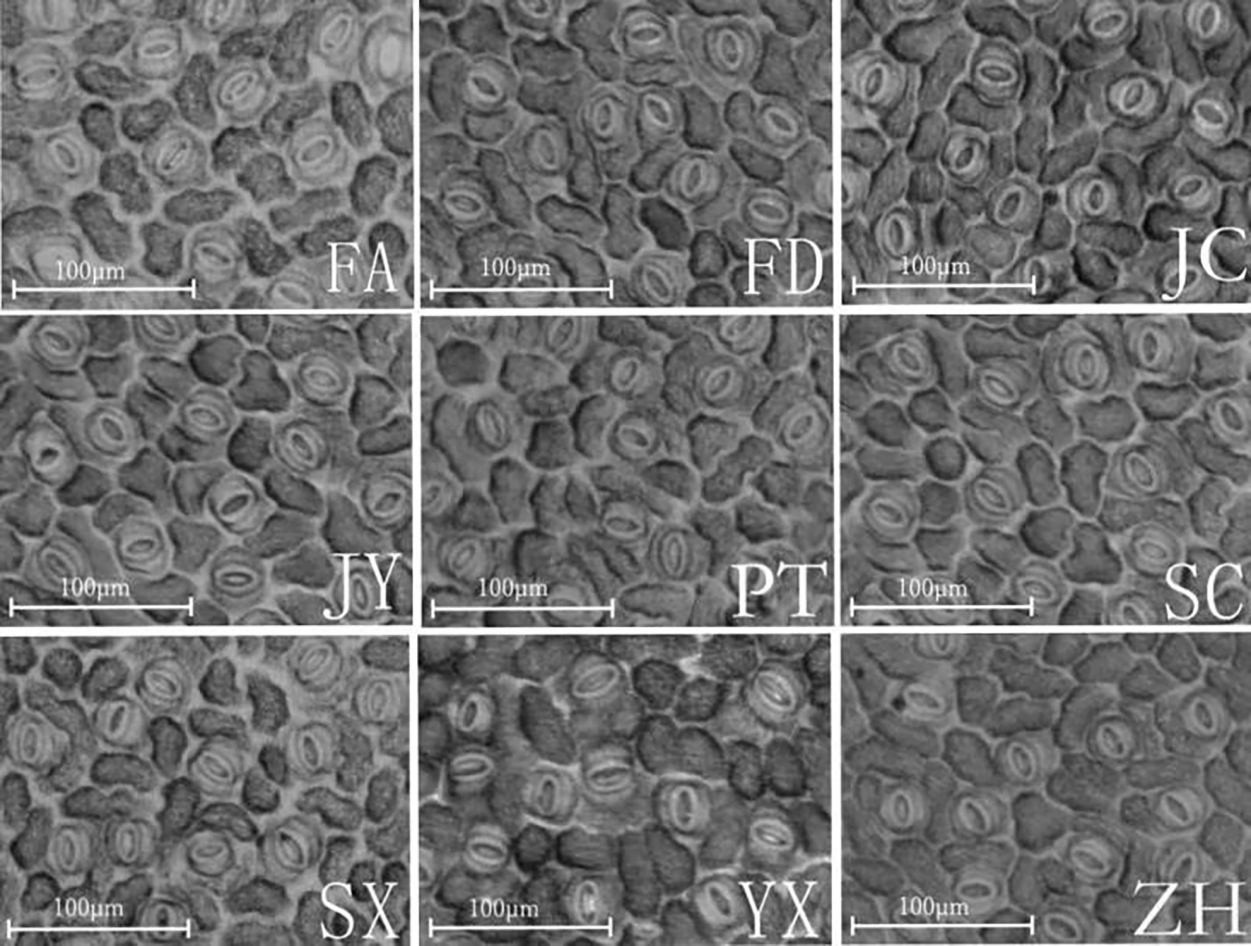
Leaf epidermis stomatal morphology for nine *A*. *montana* provenances. FA-Fuan, FD-Fuding, JC-Jiaocheng, PT-Putian, SX-Shaxian, SC-Shunchang, YX-Youxi, JY-Jianyang, ZH-Zhenghe.

Slides were observed using a stereomicroscope (Nikon SMZ18) and the stomatal characteristics were analyzed and pictured using NIS Element 7.0. The measured stomatal indexes included density, area, perimeter, long axis length, and short axis length. For each provenance, 10 slides were selected and three random pictures were obtained from each slide. Next, the area, perimeter, long axis length, and short axis length of 7–10 randomly selected stomata were measured in each picture. The number of stomata in each picture was counted and the area of each picture was measured using Image-Pro Plus to calculate the stomatal density. Stomatal characteristics are shown in Fig 1.

#### Data analysis

Data were processed using Excel 2016 and the variance analysis, correlation analysis, and cluster analysis of featured indexes of stomata were conducted using SPSS19.0. In order to further cluster provenances based on stomata density, multidimensional scaling of different *A*. *montana* stomatal characteristic parameters was done using Hierarchical Cluster Analysis Between Groups Linkage available in SPSS. In this method Euclidean distance, which was set to 5, was used to create a dissimilarity matrix.

## Results and analysis

### Characteristic analysis of stomata and stomatal apparatus

Microscopic observation of stoma morphology in different *A*. *montana* provenances is shown in Fig 1. In terms of stoma distribution, leaf stomata in all nine *A*. *montana* provenances were irregularly arranged with no fixed direction, stomata occurred infrequently in veins, and stoma distribution was sparser on both sides of veins when compared with other areas. Regarding stoma morphology, leaf stomata in all nine *A*. *montana* provenances were long and oval shaped with obvious sinks. Ridges could be observed through the stoma, with stoma ridges of stomata from different provenances or of different openness found to be different. Stomata and epidermal cells were in the same plane. Guard cells were regularly arranged, kidney-shaped, arranged symmetrically on both sides of stomata, and protruded relative to epidermal cells; the thickness of guard cell inner walls also differed. Finally, in terms of stomatal apparatus characteristics, oval stomatal apparatus were irregularly arranged and were surrounded by 3–5 epidermal cells. Epidermal cells were mainly long quadrilateral shapes with wave-like bending anticlinal walls; epidermal cell anticlinal walls were more obviously bent in Jiaocheng, Jianyang, and Zhenghe provenances.

### Analysis of stoma morphological parameters

Analysis results (Table 2) showed that the stomatal densities of tested provenances ranged from 224.16–307.10 stomata per mm^2^, with Jianyang provenance showing the highest density followed by Zhenghe provenance, and Jiaocheng provenance showing the lowest density. Meanwhile, stomatal densities differed significantly between Jiaocheng, Putian, Youxi, Jianyang, and Zhenghe provenances (P<0.05), with Jiaocheng provenance showing the highest variation coefficient of 20.15%. Stomatal densities of tested provenances differed significantly (P<0.05).

**Table 2:** Comparison of average and coefficient of variation (CV) stoma parameters of *A*. *montana* leaves.

Species	Density		Area		Perimeter		Long Axis		Short Axis		Average CV/%
	Average/mm^2^	CV/%	Average/μm^2^	CV/%	Average/μm	CV/%	Average/μm	CV/%	Average/μm	CV/%	
FA	282.43±30.12b	10.66	99.03±28.25d	28.79	41.32±5.36f	12.97	18.44±2.30d	12.49	7.46±1.29d	17.38	16.46
FD	266.21±25.44b	9.55	89.90±17.69c	19.68	39.21±3.78d	9.63	17.48±1.71b	9.78	7.23±0.92cd	12.71	12.27
JC	224.16±45.17a	20.15	97.18±29.70d	30.56	40.53±6.07ef	14.98	18.05±2.69cd	14.9	7.36±1.28d	17.45	19.6
PT	276.40±26.73b	9.67	98.88±33.39d	33.77	39.96±7.51de	18.79	17.53±3.24bc	18.48	7.39±1.59d	21.57	20.46
SX	303.01±37.52c	12.38	83.08±16.95ab	20.4	37.02±3.78ab	10.21	16.28±1.69a	10.41	6.90±0.85b	12.35	13.15
SC	281.68±37.61bc	13.35	99.76±26.46d	26.52	41.06±5.27ef	12.83	18.21±2.34d	12.85	7.35±1.05d	14.28	15.97
YX	264.66±26.63b	10.06	90.38±19.26c	21.31	39.07±4.05cd	10.37	17.30±1.81b	10.48	7.07±0.85bc	12.07	12.86
JY	307.10±29.97c	9.76	88.44±19.73bc	22.31	37.84±4.34bc	11.47	16.46±1.96a	11.88	7.03±0.84bc	11.95	13.47
ZH	303.43±27.86c	9.18	80.42±14.13a	17.57	36.39±3.39a	9.31	15.89±1.54a	9.71	6.53±0.67a	10.26	11.21

Average stomatal area of the tested provenances was 91.83 mm^2^. Shunchang provenance showed the largest stomatal area of 99.76 mm^2^ with a variation coefficient of 26.52% while Zhenghe provenance showed the smallest stomatal area of 80.42 mm^2^ with a variation coefficient of 17.57%. The stomatal areas of Fu’an, Jiaocheng, Putian, and Shunchang provenances differed only slightly.

Ranging from 36.39 to 41.3 μm, stomatal perimeters differed significantly between tested provenances (P<0.05). Fu’an and Zhenghe provenances showed the largest and smallest stomatal perimeters, respectively, while Fuding and Youxi provenances showed similar stomatal perimeters of 39.21 and 39.07 μm, respectively. Putian and Zhenghe provenances showed the largest and smallest variation coefficients of 18.79% and 9.31%, respectively.

In tested provenances, Fu’an provenance showed the longest long and short axis lengths, which were 18.44 and 7.46 μm, respectively while Zhenghe provenance showed the shortest long and short axis lengths, which were 15.89 and 6.53 μm, respectively. The variation coefficients of stomatal long axis ranged from 9.71–18.48% and the variation coefficients of stomatal short axis ranged from 10.26–21.57%.

### Correlation analysis of stomatal characteristic parameters

The correlation analysis of leaf stomatal characteristic parameters in different *A*. *montana* provenances is shown in Table 3. Pearson correlation analysis of leaf stomatal characteristic parameters (density, area, perimeter, long axis, and short axis) in nine *A*. *montana* provenances showed that leaf stomatal density was significantly negatively correlated with stomatal area, perimeter, long axis length, and short axis length (P<0.01); that stomatal area was significantly positively correlated with stomatal perimeter and long axis length (P<0.01); and that stomatal long axis length was significantly positively correlated with stomatal short axis length (P<0.01).

**Table 3:** Correlation analysis of stoma parameter of *A*. *Montana* leaves.

Parameter	Correlation coefficient between various characteristic parameters				
	Density	Area	Perimeter	Long Axis	Short Axis
Density	1				
Area	-0.301**	1			
Perimeter	-0.348**	0.960**	1		
Long	-0.388**	0.912**	0.987**	1	
Short	-0.262**	0.927*	0.831**	0.754**	1

### Cluster analysis based on stomatal characteristic parameters

**Fig 2:**
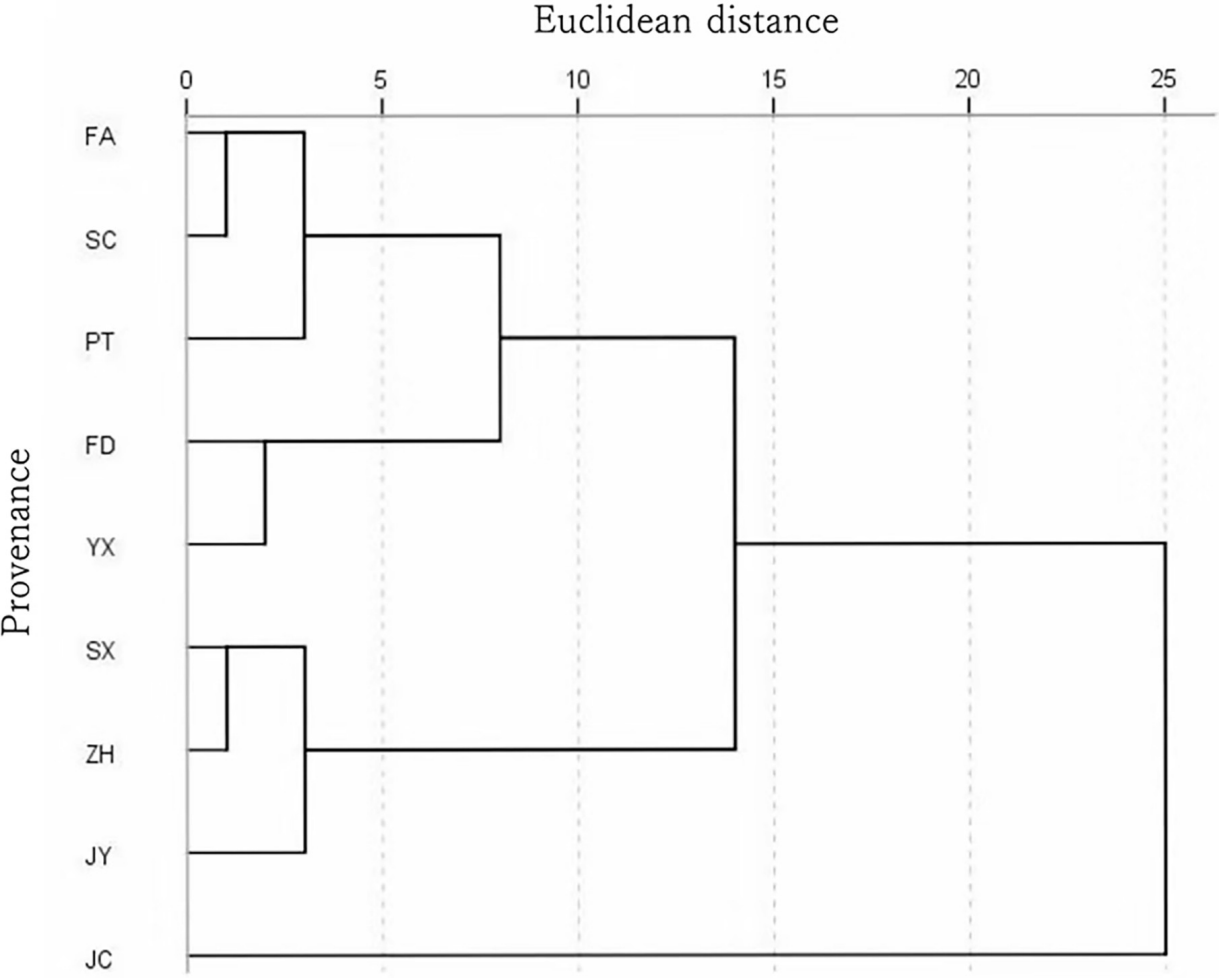
Cluster analysis of nine kinds of *A*. *montana* based on stomata parameters.

Taking five *A*. *montana* stomatal characteristic parameters as variables, cluster analysis was conducted for nine different *A*. *montana* provenances using the method of Hierarchical Cluster Analysis Between Groups Linkage (Fig 2). Results showed that, when Euclidean distance was set to 5, nine *A*. *montana* provenances could be classified into four groups. The first group, which included Fu’an, Shunchang, and Putian provenances, had similar average leaf stomatal densities, areas, and perimeters (276.40–282.43 per mm^2^, 98.88–99.76 μm^2^, and 39.96–41.32 μm, respectively), representing medium densities and large areas and perimeters. The second group, which included Fuding and Youxi provenances, had low stomatal densities ranging from 264.66 to 266.21 per mm^2^, and medium areas and perimeters. The third group, which included Jianyang, Zhenghe, and Shaxian provenances, had the smallest stomatal areas and perimeters (80.49–88.44 μm and 36.39–37.84 μm, respectively) and the highest densities (303.01–307.10 per mm^2^). With the lowest stomatal density (224.16 per mm^2^) and relatively high other parameters, Jiaocheng alone comprised the fourth group.

## Discussion and conclusions

Many factors modulate foliar stomata including abiotic factors such as temperature, moisture, radiation, carbon dioxide in the atmosphere and humidity and nutrient in the soil, as well as plants’ morphological and anatomical factors such as foliar architecture and position, plants’ photosynthetic and transpiration traits [23,24,25,26,27]. In our study, different *A*. *montana* provenances were planted in the same site to reduce environmental variation. Also, leaves from generally the same position of the plants were sampled and stomata distributing in consistent areas of adaxial sides were examined to reduce sampling errors. It’s true that the relative positions might still slightly differ because of the individual morphological differences in plants and leaves, however, the difference was minor.

Significant differences existed in stomatal characteristic parameters from different *A*. *montana* provenances, consistent with conclusions drawn by Shanna Wen et al. regarding *Manglietia conifer Dandy* leaf micromorphological characteristics (stomatal density, area, width, and length) [28]. Yanhua Zhu et al. found that growth environment significantly affected micromorphological features of plant leaves [29], suggesting that the stomatal characteristic parameter differences between different *A*. *montana* provenances might be affected by both genetic variation and environmental factors. A study by Rongbo Jiang showed that the wider a tree species was distributed, the bigger their genetic variation was, as well as their phenotypic and physiological differences. Furthermore, genetic variation was closely correlated with the complexity of their growth environment [30]. In this study, the variation coefficients of the leaf stomata of different *A*. *montana* provenances differed significantly, with a comparison of the average variation coefficient of each characteristic showing that the Putian provenance had the highest average stomatal characteristic variation coefficient of 20.46% and the Zhenghe provenance the lowest coefficient of 11.21%. These indicated that different provenances of the same plant species had different stomatal adaptability under the same growth environment. In terms of environmental changes, low variability means the plant has stronger self-regulation and environmental adaptability and better leaf stomata density stability. The Zhenghe, Fuding, Youxi, Shaxian, and Jianyang provenances, therefore, possessed better stomatal density stabilities that might be associated with genetic factors.

Casson [31] showed that stomatal density and size directly affected the transpiration and photosynthesis rates of plant leaves and that the key factors associated with plant environmental adaption were two contradictory characteristics; the adjustment of stomatal movement and optimization of stomatal density and size. Thus, correlation analysis of stomatal characteristics was of great importance. According to several studies, stomatal area, perimeter, long axis length, and short axis length are closely related to stomatal density. Observation of wheat leaf stomata by Weiyue Chen[32] showed that leaf stomatal density was significantly negatively correlated with the stomatal long axis and short axis length. In this study, consistent with other reports, stomatal density was significantly negatively correlated with stomatal perimeter, area, long axis length, and short axis length.

Comparison of five stomatal characteristics (stomatal density, area, perimeter, long axis length, and short axis length) in leaves of nine different *A*. *montana* provenances and cluster analysis based on those characteristic parameters showed that, when the Euclidean distance was set to 5, these nine *A*. *montana* provenances could be classified into four groups. Most had high stomatal densities that were generally accompanied by smaller stomatal sizes. In terms of environmental changes, smaller stomata had shorter reaction times and faster open/close times compared with larger ones, meaning that the combination of high density and small size improved the stomatal conductance under benign environments and optimized the CO_2_ diffusion rate to decrease the stomatal conductance under adverse environments [33,34]. This indicated that most *A*. *montana* provenances had strong environmental adaptabilities, in particular, the Jianyang, Zhenghe, and Shaxian provenances.

To summarize, analysis of stomata showed the *A*. *montana*, Jianyang, Zhenghe, Fuding, and Shaxian provenances were better transplant choices when considering their stomatal stabilities and stomatal environmental adaptabilities, thereby facilitating the selection of better *A*. *montana* provenances. Currently, there is no information about the effect of stomatal features on some other foliar functional traits such as photosynthesis, water balance, and construction cost. This might be a future challenge.

## Supporting information

S1 Dataset Stomota traits data of different provenances. (XLSX)

S2 Dataset Stomata density data. (XLSX)

S1 File Certificate of editing. (PDF)

## References

[1] Taylor SH, Franks PJ, Hulme SP, Springs E, Christin PA. Photosynthetic pathway and ecological adapation stomatal trait diversity amongst grasses. *New Phytol*. 2010;193 (2): 387–396. 10.1111/j.1469-8137.2011.03935.x 22040513

[2] Franks PJ, Beerling DJ. Maxiumu leaf conductance driven by CO_2_ effects on stomatal size and density over geologic time. *Proc Natl Acad Sci USA*.2009; 106 (25): 10343–10347. 10.1073/pnas.0904209106 19506250 PMC2693183

[3] Ma D. Stomates [M]. Beijing: Scince Press, 1987:10–30.

[4] Hetherington AM, Woodward FI. The role of stomata in sensing and driving environmental change. *Nature*.2003;424 (2): 1737–1748. 10.1038/nature01843 12931178

[5] Beghin T, Cope JS, Remagnino P. Shape and texture based plant leaf classification [M]. / /Blanc-Talon J, Bone D, Philips W. Advanced concepts for intelligent vision systems Berlin Heidelberg: Springer 2010;64:345–353.

[6] Jia CH, Zhang L, Wei X, Yu J, Li M. Phenotypic Polymorphism of Litsea mollis Hemsl in West Sichuan Province. *For Res*.2015;28(6):844–850. 10.3969/j.issn.1001-1498.2015.06.013

[7] Zhu LL, Wu JC, Peng XM, Zheng YM, Zhang YP. Phenotypic Difference among Species and a Variation Type of Azadirachta. For Res.2016; 29(2):162–166. 10.3969/j.issn.1001-1498.2016.02.002

[8] Liu J, Wang l, Xing Y. Morphology structure of leafepidermis of Maloideae in Heilongjiang. *J Chin Electron Microsc Soc*.2014;3 (1):69–76.

[9] Yang CY, Shi JY, Du XG, Dong H.Studies on the stomata of apple leaves. *J Shandong Agric Univ*.1998;29(1):8–14.

[10] Li RT, Zhang YN, Tian DL. Studies on Stomata of Citrus Plant Leaves. J Fru Sci.2004;21(5):419–424. 10.3969/j.issn.1009-9980.2004.05.007

[11] Alec JJ. Paraguayan tung: an important small farmer crop diversification strategy[D]. Houghton: Michigan Technological University, 2002

[12] Lin H, Chen H, Wu CZ, Hong T, Xie AQ. Effects of Decomposition of Aleurites montana and Phyllostachys pubescences Mixed Foliage Litter on Activity of Soil Enzymes. *Chin J Appl Environ Biol*.2012;18(4):539–545. 10.3724/SP.J.1145.2012.00539

[13] Lin H, Chen H, Wu CZ, Hong T, Xie AQ.Comparison of foliar endogenous hormones in different Aleurites montana provenances and their correlation with C and N contents. *J Fujian Coll For*.2012;32(2):102–106. 10.3969/j.issn.1001-389X.2012.02.002

[14] Lin H, Chen H, Wu CZ, Chen C, Li J, Lin YM, Xie AQ. Comparison of chlorophyll fluorescent characteristics of Aleurites montana among different provenances. J Fujian Agric For Univ (Nat Sci Ed).2012;41(1):34–39.

[15] Lin H, Chen H, Wu CZ, Hong T, Chen C, Chen JZ, Liu JB, Xiao YZ. Relationship between Foliar δ13C and Environmental Factors andFoliar C and N Contents of Aleurites montana in Fujian Province. *J Nat Res*.2013;28(8):1328–1336. 10.11849/zrzyxb.2013.08.006

[16] Lin H, Chen H, Wu CZ, Hong T, Li J, Lin YM, Fan HL. Provenance Selection of Aleurites montana Based on Endogenous Hormone.Chin J Trop Crops.2012;33(11): 1937–1941.

[17] Yi XL, Wang JX, Duan ZQ. Study on the Stomatal Density and Daily Change Rule of the Wheat. Chin Agric Sci Bull.2006;22 (5):237–242. 10.3969/j.issn.1000-6850.2006.05.063

[18] Chen L C Li CS, Chaloner WG.Assessingthe potential for the stomatal characters of extant and fossil Ginkgo leaves to signal atmospheric CO_2_ change. *Am J Bot*.2001;88: 1309–1315. 11454631

[19] Xu ZZ, Zhou GS.Responses of leaf stomatal density towater status and its relationship with photosynthesis in agrass.*J Exp Bot*.2008;59: 3317–3325. 10.1093/jxb/ern185 18648104 PMC2529243

[20] Reich PB. Leaf stomatal density and diffusive conductancein three amphistomatous hybrid poplar cultivates. *New Phytol*.1984;98:231–239.

[21] Yi XL, Wen J, Liu X. Study on leaf stoma density of 12 representative genus of Rosaceae family. Northern Frui.2008;1: 4–6.

[22] Poole I, Weyer J, Lawson T.Variations instomatal density and index:Implications of palaeo climatedrecon struction.Plan, Cell & Environ.1996;19:705–712.

[23] Massonnet C, Costes E, Rambal S, et al Stomatal regulation of photosynthesis in apple leaves: evidence for different water-use strategies between two cultivars. Ann Bot.2007;100(6):1347–1356. 10.1093/aob/mcm222 17901058 PMC2759240

[24] Singh S K, Reddy K R. Regulation of photosynthesis, fluorescence, stomatal conductance and water-use efficiency of cowpea (Vigna unguiculata [L.] Walp.) under drought. J Photochem Photobiol B, 2011;105(1):40–50. 10.1016/j.jphotobiol.2011.07.001 21820316

[25] Martins G A, Soares A M, Mello J M D, et al Stomatal density distribution patterns in leaves of the Jatobá (Hymenaea courbaril, L.). Trees.2012; 26(2):571–579.

[26] Baruch Z, Christmas M J, Breed M F, et al Leaf trait associations with environmental variation in the wide‐ranging shrub Dodonaea viscosa subsp. angustissima (Sapindaceae). Austral Ecology.2017; 42(5):1–9.

[27] Tobin R L, Kulmatiski A. Plant identity and shallow soil moisture are primary drivers of stomatal conductance in the savannas of Kruger National Park. Plos One.2018;13(1):e0191396 10.1371/journal.pone.0191396 29373605 PMC5786297

[28] Wen SN, Zhong CL, Jiang QB, Chen Y, Zhang Y, Li QY.Phenotypic Diversity Analysis of Seedling Leaf Traits of Manglietia conifera Dandy. *Bull Bot Res*.2017;37(2):291–295. 10.7525/j.issn.1673-5102.2017.02.018

[29] Zhu YH, Kang HZ, Liu CJ.Affecting factors of plant stomatal traits variability and relevant investigation methods.*Chin J Appl Ecol*.2011;22(1): 250–256. 21548316

[30] Jiang Rongbo, Liu J Jiang JM, Kong C Li YJ. Geographical Variation in Main Phenotypic Traits and Seedling Traits of Machilus thunbergii. J Northeast For Univ.2011;39(5):9–12.

[31] Casson SA, Hentherington AM.Envirnmental regulation of stomatal development.*Curr Opin Plant Biol*.2010;13(1):90–95. 10.1016/j.pbi.2009.08.005 19781980

[32] Chen WY, Liu CH, Li YY, Mi DH. Flag leaf vein traits in winter wheat varieties (lines) and their correlation with stomatal traits. *Chin J Ecol*.2014;33(7):1839–1846.

[33] Hetherington AM, Woodward FI. The role of stomata in sensing and driving environmental change. *Nature*.2003;424(6951): 901–908. 10.1038/nature01843 ,DOI:12931178

[34] Drake PL, Froend RH, Franks PJ.Smaller,faster stomata:scaling of stomatal size,rate of response, and stomatal conductance.J Exper Bot.2013;64(2):495–505. 23264516 10.1093/jxb/ers347 PMC3542046

