# Supplementary material for: Characteristics and correlations of leaf stomata in different Aleurites montana provenances
Source: PLoS One. 2018 Dec 18;13(12):e0208899. doi: 10.1371/journal.pone.0208899 (PMC6298671; doi:10.1371/journal.pone.0208899)
Supplement: S1 File — (PDF) [file pone.0208899.s003.pdf]

Certificate of English Editing

**Date of Issue**	13 August 2018

About the manuscript:

**Title**	Characteristics and correlations of leaf stomata in different Aleurites montana provenances
**First Author**	Hong Tao
**Affiliation**	Fujian Agriculture and Forestry University
**Date of editing**	17 April 2018

About the editor:

Editor

Emma Tacken

2013 - PhD Biology - University of Auckland

Plant Molecular Biologist focused on characterizing regulatory and developmental pathways in model and non-model plant species

Full profile

**Certificate issued by**	Benjamin Shaw<br>Director<br><br>Liwen Bianji (Edanz Group China)

While this certificate confirms the authors have used Edanz's editing services, we cannot guarantee that additional changes have not been made after our edits. It is the author's responsibility to ensure any unclear sentences in the manuscript are clarified for the Edanz editor.

Liwen Bianji (Edanz Group China)
Interchina Commercial Building, 1112A
No 33. Dengshikou Street, Dongcheng District, Beijing, P.C. 100006, China
Phone +86-10-6528-0877 Fax +86-10-6528-0834 Email editing@liwenbianji.cn
